# Behavioral Response of *Leptoglossus zonatus* (Heteroptera: Coreidae) to Stimuli Based on Colors and its Aggregation Pheromone

**DOI:** 10.3390/insects9030091

**Published:** 2018-07-26

**Authors:** Sandra Lisbeth Franco-Archundia, Agustín Jesús Gonzaga-Segura, Alfredo Jiménez-Pérez, Víctor Rogelio Castrejón-Gómez

**Affiliations:** Centro de Desarrollo de Productos Bióticos (CEPROBI), Instituto Politécnico Nacional. Carretera Yautepec, Jojutla, Km. 6 calle Ceprobi No.8, San Isidro, Yautepec, Morelos 62739, Mexico; sandy182_@hotmail.com (S.L.F.-A.); jgonzagas@live.com (A.J.G.-S.); aljimenez@ipn.mx (A.J.-P.)

**Keywords:** attraction, traps, volatiles, pheromone, pest, colors, communication

## Abstract

The leaf-footed bug *Leptoglossus zonatus* (Dallas) (Heteroptera: Coreidae) is an important pest in the Americas. However, no preference of colors, sexual behavior nor aggregation pheromone has been reported, which can be used for detection, monitoring, and control purposes. In the laboratory we tested the attractiveness of white, violet, blue, green, yellow, and orange color to nymphs and adults (mated and unmated) and found that most adults and nymphs were attracted to and remained longer on blue and green colored cards than the other colors tested. We found that couples may remain in copula ≈185 min and mate ≈20 times in a 60 d period with a similar number of matings during the scotophase and the photophase. Sexual behavior consists of six patterns: grooming, abdomen movement, antenna movement, antennation, mounting, and mating. In a Y-tube olfactometer, 80 and 62.5% of the adults tested were attracted to a hexane-extract of the volatiles released by 40 males and 40 females, respectively. This is the first report of the biological evidence of an aggregation pheromone in this bug.

## 1. Introduction

In Heteroptera, communication involves acoustic (vibration), chemical (pheromones and plant volatiles) and visual (light and color) signals [1,2,3] to find mates, food, oviposition sites, shelter, etc. Many heteropteran nymphs produce and emit aggregation and sex pheromones through their dorso-abdominal gland and adults by their metathoracic glands [4,5,6,7,8], which are detected by the antennal sensilla of conspecifics [9]. Visual and olfactory stimuli attractive to insects can be combined to develop new management tools. Colored traps take advantage of the attraction that color or wavelength have on specific insects. Different authors have shown that yellow traps catch *Frankliniella occidentalis* (Pergande) (Thysanoptera) [10], *Chaetocnema pulicaria* Melsheimer (Chrysomelidae) [11], *Trialeurodes vaporariorum* Westwood and *Bemisia tabaci* (Gennadius) (Hemiptera) [12,13,14], the blue light traps catch *Lycorma delicatula* White (Hemiptera) [15] and the blue traps are used for mass trapping of some thrips [16].

Olfactory stimuli, like sex or aggregation pheromones or plant volatiles, can be integrated into colored traps for a more specific, and attractive trap. Traps including color and olfactory stimuli have been developed for the brown marmorated stink bug (BMSB) *Halyomorpha halys* (Stål) [17,18,19], the Brown Winged Stink bug *Plautia crossota stali* Scott [20,21], and the Shield bug *Glaucias subpunctatus* (Wotlk) (Pentatomidae) [20].

The leaf-footed bug *L. zonatus* is widely distributed across America [22] and is an effective vector of the pathogenic yeast *Nematospora coryli* Peglion (Nematosporaceae: Hemiascomycetidae) to oranges [23]. The adults and nymphs feed on many important crops, like maize, sorghum [22], pomegranate [24], citrus [25,26], and *Jatropha curcas* L. (Malpighiales: Euphorbiaceae), an important source of biodiesel [27,28]. In 2003, the metathoracic scent gland of males and females of this insect was described [29]. Despite its importance as a vector and crop pest, many aspects of its biology remain unknown. There is no information on its color preference or the possibility of using its sex or aggregation pheromone in traps for monitoring or control purposes. This may be because, as in many other Hemiptera, *L. zonatus* release an alarm pheromone [30] making it difficult to identify its sex or aggregation pheromone [31,32]. Leal et al. [30] reported nymphs and adults aggregate on maize crops. Therefore, we conducted a series of studies aimed at determining color preferences, describing basic elements of the sexual behavior, and investigating the presence of an aggregation pheromone to lay the foundation for the development of an efficient capture method for *L. zonatus*.

## 2. Materials and Methods

### 2.1. Insects

Males and females were collected on pomegranate trees at Cuautla (18°50′24″ N 98°56′49″ W), Yecapixtla (18°52′40″ N 98°51′33″ W) and Yautepec (18°53′03″ N 99°04′20″ W) at Morelos state, Mexico. Insects were identified according to Brailovsky and Barrera [33], and voucher specimens were deposited at the Chemical Ecology lab at Ceprobi, IPN. Males and females were kept in acrylic transparent containers (25 width × 25 length × 25 height cm) covered with a mesh. Adults and nymphs were fed with corn cob (milky state), green beans, and water. Most eggs were collected from the walls and some from the mesh and placed in the same type of container just described. Colony and experimental conditions were 24.5 ± 2.8 °C and 55.7 ± 19.2% RH. White light was provided by four sets of 2-tubes (20 watts, LED T8, SL^®^, Philips^®^ Naucalpan de Juarez, Mexico).

### 2.2. The Response of Nymphs and Adults of Leptoglossus zonatus to Different Colors

Independent bioassays were used for nymphs and adults. Assays on nymphs were carried out in acrylic circular arenas (15 cm diameter by 10 cm height) and divided into six equal quadrants, each of which had a different colored card: white, violet, blue, green, yellow, or orange [15]. Adults were tested in a transparent acrylic arena (130 cm width × 250 cm length × 50 cm height). Six cards (28 × 21 cm each) with the colors mentioned above were stuck to the walls of the arena. The colored cards that were evaluated in the nymph and adult trials were created with ColorSchemer Studio software on an RGB (Green, Red, and Blue) format and were printed on matte paper. White (RGB = 255/255/255), violet (RGB = 148/0/211; λ = 420 nm); Blue (RGB = 0/0/225, λ = 440 nm); green (RGB = 0/225/0, λ = 510 nm), yellow (RGB = 0/225/225, λ = 580 nm) and orange (RGB = 255/140/0; λ = 615 nm) cards were tested in the arena. The white card was used as a control. In both bioassays, the arenas were cleaned with ethanol after testing each insect to avoid any pheromone related effects. In assays on adults, the colored card position in the arena was randomly assigned every morning. In nymph trials, the circular arena was turned clockwise 90 degrees. Insects were isolated in a transparent plastic container (4 cm diameter by 4 cm height) covered with a plastic lid and introduced in the bioassay room 1 h prior to experiment. A nymph or an adult was placed at the center of the arenas as appropriate, and the number of times and the time spent in each color was recorded for 10 min. Forty nymphs of the 4th and 40 nymphs of the 5th instar and a total of 160 adults (40 virgin females, 40 mated females, 40 virgin males, and 40 mated males) were tested.

### 2.3. Sexual Activity of Leptoglossus zonatus

The objective of this experiment was to know at what age these insects reach sexual maturity and the time of the day when most matings occurred. Two rooms were set up with different photoperiods with 2 d old couples in each room. To obtain the data of the scotophase and the photophase in the same period of observations, the scotophase in one room was set between 19:00–06:00 h and the other room between 08:00–19:00 h. A male and a female were confined in a cylindrical plastic transparent container (9 cm diameter by 15 cm height). Preliminary observations allowed us to determine that matings take more than 2 h, therefore the observations were every 2 h (in the light or dark period) for 24 h every other day until insects were 60 d old. At the end of each observation period (24 h), insects were kept individually in cylindrical containers (5 cm diameter by 8 cm height) to avoid mating and were introduced into the observation cage the next observation day. Sexual activity during the scotophase was recorded with the aid of three red lights (40 watts, GE^®^). A corn cob and water were offered at all times. A total of 20 pairs were observed.

### 2.4. Mating Behavior of Leptoglossus zonatus

A 25 d old male and female were placed at a distance of 30 cm at the beginning of the observations in an acrylic transparent box (45 width × 90 length × 60 height cm). Preliminary observations allowed us to determine that a calling peak occurs around 14 h in the photophase, consequently the observations were made between 12:30 and 14:30 h and their sexual behavior was recorded for an hour. Observed behaviors were tabulated in first-order contingency tables and an ethogram was designed. Only those frequencies greater than expected were included in the ethogram [34]. A total of 30 pairs were observed.

### 2.5. Active Volatile Collection from Conspecifics

Both genders have a metathoracic scent gland [28], so we collected the volatiles released by 18–25 d old virgin insects: 40 males, 40 females, and 20 males + 20 females were used with three solvents (hexane, ethyl acetate/ethanol 3:1 or methanol) for each group of insects. Insects were placed into a glass container (41 cm length × 10.5 cm internal diameter) with an air inlet and outlet. Air was cleaned by an activated granulated charcoal filter and pulled through the container at 500 mL/min (flowmeter Cole Parmer PMR 1-0110293, Cole-Parmer Instrument Company, Vernon Hills, IL, USA). Volatile extraction was divided into two sets of 12 h each one (from 20:00 h to 08:00 h and 08:00 h to 20:00 h of the next day). Emitted compounds were trapped on a Pasteur pipette packed with 125 mg of SuperQ^®^ (Altech Associates, Inc., Deerfield, IL, USA). Captured volatiles were eluted with 1.5 mL HPLC grade either hexane, ethyl acetate/ethanol 3:1 or methanol as appropriate in 2 mL dark vials for each group of insects in which volatiles were collected. Each extract was concentrated to 200 μL (equivalent to 0.2 insects/μL) on a nitrogen flow and kept at −20 °C until needed.

### 2.6. Behavioral Response of Leptoglossus zonatus to Conspecific Volatiles

A Y-tube olfactometer was used to test the response of *L. zonatus* to its conspecific volatiles (3.5-cm inner diameter clear glass tube with a 19-cm-long central tube and two 12-cm-long lateral arms with a 90° angle at the Y-junction). The lure to test (2 μL) was applied to a 1 cm^2^ piece of Whatman No. 1 filter paper and placed at the end of a lateral arm. The other arm of the tube functioned as the control (clean air). A continuous flow of 0.5 L/m charcoal purified air was pumped into each arm of the olfactometer. Insects were isolated in plastic transparent containers (4 cm diameter by 4 cm height) and placed in the bioassay room 2 h prior to testing. An insect was placed at the end of the central tube and was observed for 5 min. Any insect reaching 2 cm inside any of the arms of the olfactometer was considered as a response (modified from [35]). A total of 9 extracts: three groups of insects × three different solvents mentioned above were evaluated. An additional three led lamps providing 2 watts of white light (0.8–1 lux) were placed above the olfactometer. Previously the highest percentage of mating had been observed at 14 h of the photophase, therefore the bioassays were performed between 12:00 and 16:00 h.

### 2.7. Statistical Analysis

Adult and nymphal color preferences were analyzed with a G test and a non-parametric analysis of variance (ANOVA) followed by a Tukey test. A z test was used to compare the proportion of total insects attracted to color (wavelengths). Nymph latency on colors was analyzed with a two-way ANOVA (color and nymphal stage as factors) and a Fisher test. Adult latency on colors was analyzed with a three-way ANOVA (color, gender, and mating status as factors) followed by a Fisher test. The number of matings recorded during the photophase and the scotophase were compared with a Mann–Whitney test. Choice experiments were analyzed with a *χ*^2^. Rejection probability was set at 0.05. Statistical analysis was carried out on SigmaPlot 12.5 (Systat Software Inc., Chicago, IL, USA).

## 3. Results

### 3.1. Color Preference

A small number of insects were recorded at the yellow and orange color cards, so these data were pooled together. Data of insects recorded in the white, yellow/orange, or violet color trials were not included in the analysis because the minimum expected frequency was <5, invalidating the G test, however, these insects represent 30% of the insects in the experiment. Almost 50% more insects were registered at the blue than to the green color, nevertheless, this difference was not significant (*χ*^2^ = 4.94, df = 5, *p* > 0.05). Similarly, no difference was observed in the number of virgin and mated females (*χ*^2^ = 0.001, df = 1, *p* > 0.5), the number of virgin or mated males (*χ*^2^ = 1.0, df = 1, *p* > 0.5) or the number of virgin (males + females) or mated (males + females) (*χ*^2^ = 0.57, df = 1, *p* > 0.5) adults recorded at the blue or green color. Similar number of 4th and 5th instar nymphs (*χ*^2^ = 0.75, df = 1, *p* > 0.5) were registered to the previously mentioned colors and no difference was observed in the number of immature insects and adults (*χ*^2^ = 2.5, df = 1, *p* > 0.5) recorded to the blue or green color.

**Figure 1:**
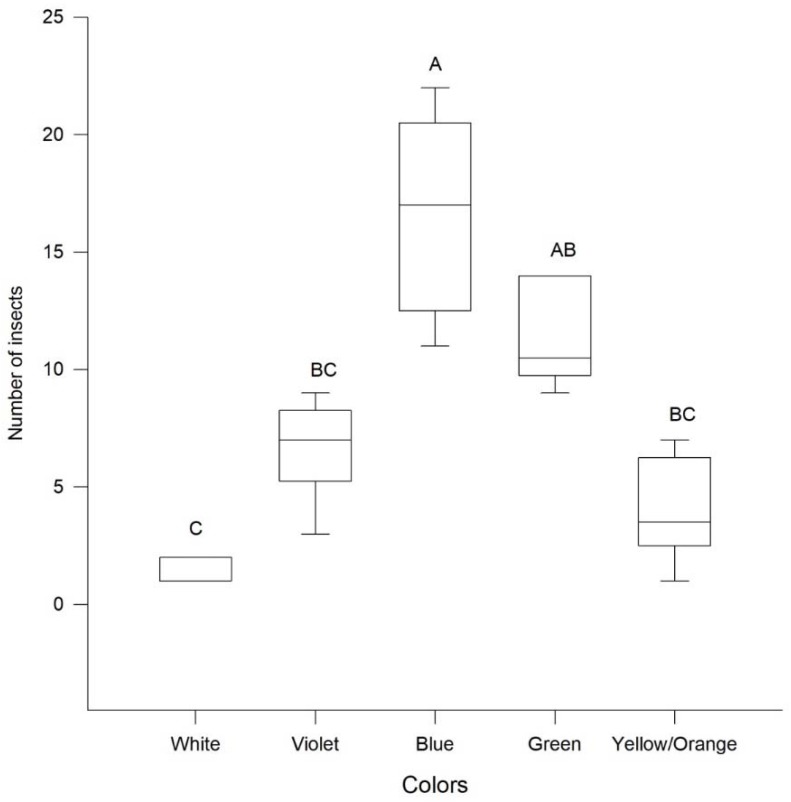
Number of insects (adults + nymphs) of *Leptoglossus zonatus* observed at different colors (KW, H = 25.87, df = 5, *p* > 0.05). Box plots represent the 1st quartile, median and 3rd quartile, respectively; whiskers represent the 5 and 95% of the data. Bars topped by the same letter are not significantly different (Tukey, *p* > 0.05).

More insects (adults and nymphs) were observed at the blue, green, and violet (λ= 420–510 nm) than at the yellow/orange (λ = 580–615 nm) and white color (KW test, H = 25.87, df = 4, *p* > 0.001) (Figure 1). A significantly larger proportion (86%) of insects were observed either to the blue, green and violet than to the yellow/orange and white color (*z*-test = 10.87, *p* > 0.001).

### 3.2. The Response of Nymphs and Adults of Leptoglossus zonatus to Different Colors

**Figure 2:**
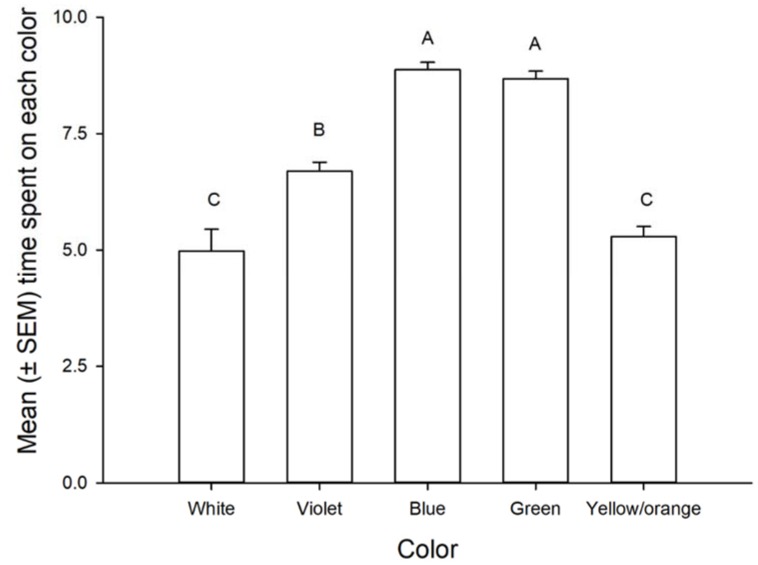
Mean (±SEM) minutes spent on each color by nymphs of *Leptoglossus zonatus*. Bars followed by the same letter are not significantly different. Error bars are SEM (Fisher, *p* > 0.05).

Nymphs spent significantly more time on the blue, green and violet colored cards than on yellow/orange or white (control) cards (F = 69.61, df = 4,70, *p* < 0.001 for color; F = 1.41, df = 1,70, *p* = 0.7 for nymphal stage and F = 2.0, df = 4,70, *p* = 0.1 for the interaction color × nymphal stage) (Figure 2).

**Figure 3:**
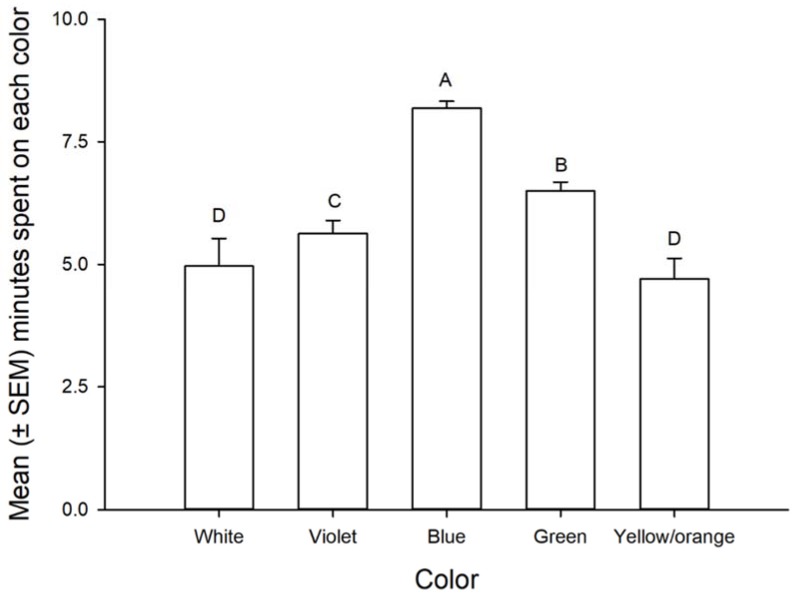
Mean (±SEM) minutes spent on each color by adults of *Leptoglossus zonatus*. Bars followed by the same letter are not significantly different. Error bars are SEM (Fisher, *p* > 0.05).

**Figure 4:**
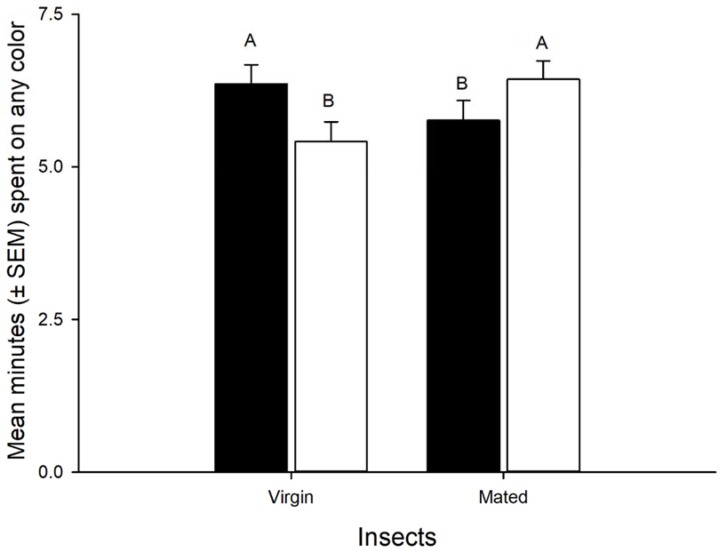
Mean (±SEM) minutes spent on any color by virgin or mated, females (solid bars), and males (empty bars). Bars topped by the same letter are not significantly different. Error bars are SEM (Fisher, *p* > 0.05).

Adult time spent on color cards was influenced by color (F = 37.21, df = 4,140, *p* < 0.001) but not by gender (F = 0.190, df = 1,140, *p* > 0.05) or mating (F = 0.44, df = 1,140, *p* > 0.05). However, the interaction gender × mating was significant (F = 6.63, df = 1,140, *p* = 0.01). Adults significantly spent more time on blue than green followed by violet and these were significantly different from the yellow/orange and the white (control) (Figure 3). Virgin females spent more time on the colors than virgin males. However, the opposite was observed for mated insects and females spent less time on the colors than males (Figure 4).

### 3.3. Mating Activity of Leptoglossus zonatus

**Figure 5:**
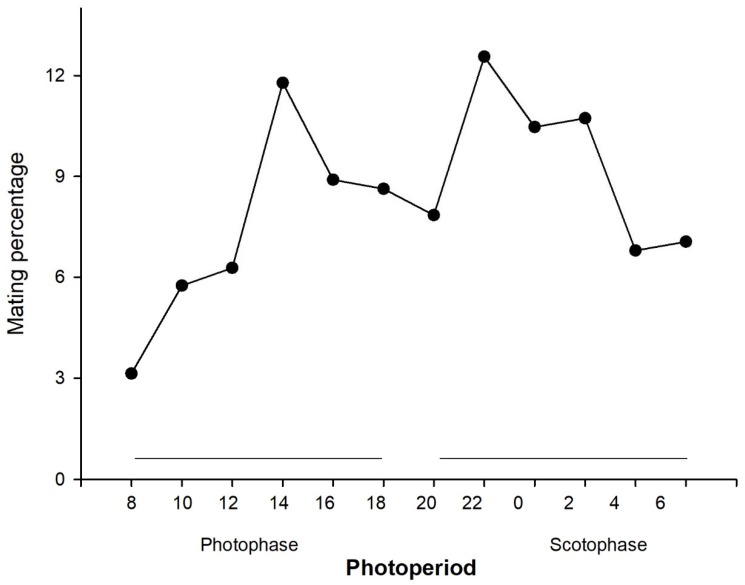
Percentage of *Leptoglossus zonatus* mating during the photophase and scotophase during 60 d of observation. Lights went off and on at 19:00 and 06:00 h, respectively. Horizontal lines represent the length of the scotophase and photophase.

A total of 382 matings were observed during the 60 d observation period with an average of 19.1 (SEM = 2.32) matings/couple. The first mating was observed when insects were 25 ± 1.83 d old (mean ± SEM) and lasted for 185 ± 20.14 min (mean ± SD). Similar numbers of matings were observed during the scotophase (median, 25–75% = 35, 26.7–42.7) and the photophase (median, 25–75% = 28.5, 19.5–36) (Mann–Whitney U Statistic = 11.000, *p* > 0.05). Two peaks of sexual activity were observed: one in the photophase at 14:00 h and the other at 22:00 h into the scotophase (Figure 5).

### 3.4. Mating Behavior of Leptoglossus zonatus

**Figure 6:**
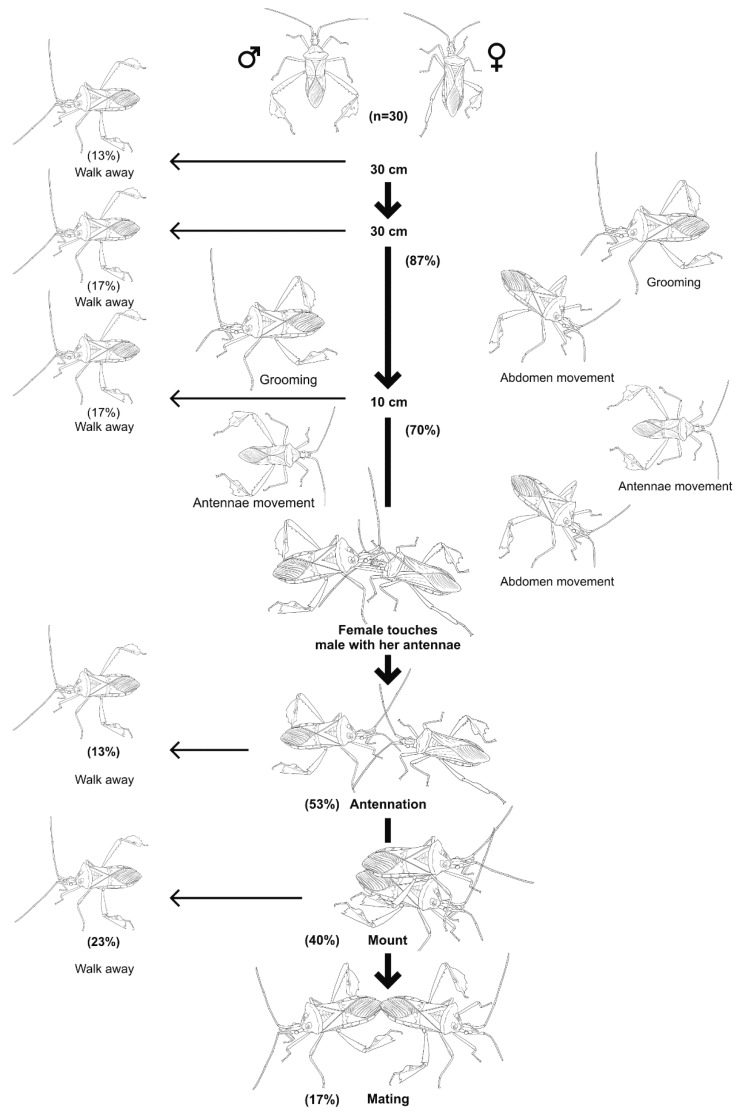
Ethogram of the mating behavior of *Leptoglossus zonatus* in laboratory conditions. Recordings were made during the photophase.

A total of 6 behaviors were recorded: grooming, abdomen movement, antennal movement, insects touching each other with the antennae (antennation), mounting and mating (Figure 6). Insects groomed and moved their antennae and moved randomly when they were more than 30 cm apart. At closer range (less than 30 cm apart) males would stop grooming and move their antennae when females moved their abdomen laterally, then males moved toward females and touched them with their antennae. Females increased the frequency of the lateral movement of their abdomen and touched the males’ antennae. After female contact, males (40%) mounted the female, and both insects faced the same direction. The male continued antennation and moved his body 90° and placed his ninth abdominal segment in the female’s genitalia. If a female was receptive, she remained motionless, touched the male’s body with her antennae, and the male moved until both sexes faced different directions. If females were not receptive, she would kick the male’s body with their hind legs, shake her body and run away. Males would go after the female or simply remain still. Seventeen percent of the 30 pairs achieved mating.

### 3.5. Response to Conspecifics Volatiles

**Figure 7:**
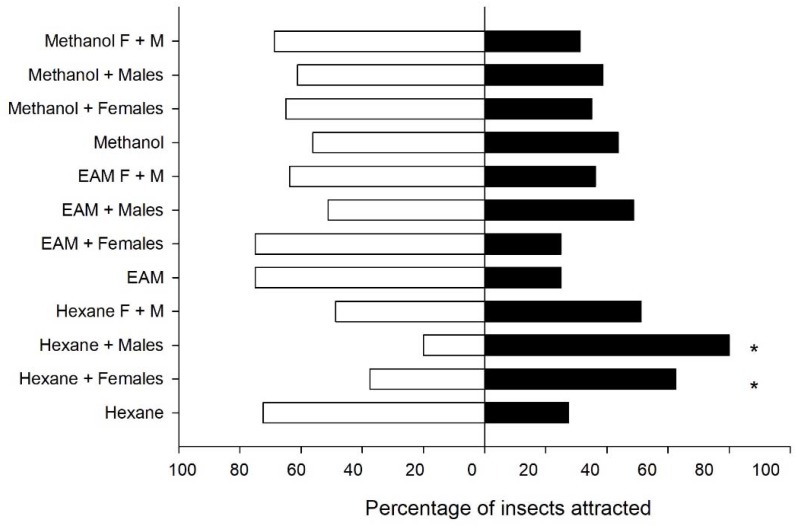
Percentage of *Leptoglossus zonatus* adults attracted to different extracts (solid bars) or air (empty bars). * indicated significant differences (binomial test *p* < 0.05). EAM = Ethyl acetate + methanol 3:1. N = 80.

Male and female response to the different lures was not significantly different (*χ*^2^ from 0 to 0.84, df = 1, *p* > 0.05) so data were pooled for further analysis. There were significant differences between the extracts and their controls (*χ*^2^ = 84.89, df = 11, *p* < 0.001). Male hexanic extract attracted more males and females than any other one followed by the female hexanic extract (*χ*^2^ = 13.41, df = 1, *p* < 0.001) (Figure 7).

## 4. Discussion

Our results show that ≈70% of adults and nymphs were attracted to the blue and green color indicating that a blue or green colored trap could be successful in trapping nymphs and adults of *L. zonatus*, as found for other species of Hemiptera. For example, adults and nymphs of the bug, *Lycorma delicatula* (White) (Fulgoridae) are attracted to the blue and green color [15]. Similarly, nymphs and adults of the Miridae *Lygus hesperus* Knight were attracted to the green color [3], and adults of the Pentatomidae *Halyomorpha halys* were attracted to green, orange, red, and yellow light. However, in this case, adult males were more attracted than any other life stage, and white light was the most attractive stimulus [36], while in our study only 3% of all the insects tested were attracted to this color. Specialist insects tend to detect wavelength related to their host, while generalist insects like *L. zonatus* respond to a broader wavelength spectrum [37].

Sexual maturity in Heteroptera seems to be related to environmental conditions (temperature, HR, and light period) and type of food available among other factors [38,39]. For example, sexual maturity on *L. zonatus* fed on corn cob (*Zea mays*) at 24.5 ± 2.8 °C, 55.7 ± 19.2% RH and 11:13 h (L:D) light period has the highest number of days reported until now. A closely related species, *Leptoglossus clypealis* (Heidemann) (Coreidae) required fewer days (16.42 ± 0.42 d, means ± SEM) to reach maturity at 27 ± 1 °C, 60 ± 10% RH and a 16:8 h (L:D) light period, fed on green beans and sunflower seeds [40]. Other Heteropterans mature earlier like *Lygocoris pabulinus* L. (Miridae), which needs 4.5 d, at 25 °C and 65% RH and 18:6 h (L:D), fed with potato leaves [39].

Matings in our study were recorded throughout the day with 55% of them during the scotophase and a peak at each light period, which is in concordance with the 55% matings reported by Wang and Millar [40] for *L. clypealis* (Coreidae). Mating activity peaks may vary among different bugs, for example, mating peaked between 1 and 8 h within the photophase in *Nysius huttoni* (White) (Lygaeidae) [41], while in *Dysdercus koenigii* (Fabricius) (Pyrrhocoridae) most matings were recorded at the last hours of the photophase [42]. Different circadian mating times may synchronize oviposition and migration with the environmental conditions, like temperature, RH, and food [41].

Like most bugs, *L. zonatus* followed a specific path to mating, which involved movement toward their possible partner, antennation, touching the females’ abdomen, mounting, and mating and is similar to the mating behavior of *L. clypealis* [40], except that the ninth abdominal segment was always visible and not concealed within the seventh abdominal segment. Antennation is a very important part of the courtship as it is widely spread among bugs, like *Murgantia histrionica* (Hahn) [43], *Euschistus conspersus* (Uhler) (Pentatomidae) [44], *Lygocoris pabulinus* L. [39] and *Nezara viridula* L. [45]. Some other behaviors may be present in other bug species, like abdomen vibration before mating in *L. pabulinus* [39] or absent, such as no female antennation in *L. clypealis* [40]. Clear evidence of male pheromone release behavior was observed in *Bagrada hilaris* (Burmeister) (Hemiptera: Pentatomidae) [46] and *L. clypealis* (Hemiptera: Pentatomidae) [40], but this behavior was not recorded in *L. zonatus*.

Our results indicate that males and females of *L. zonatus* release an aggregation pheromone. This possibility had been suggested previously as [29] reported the presence of metathoracic scent glands in both sexes but no biological evidence was available at that time. In many Heteropteran species, one or both sexes release sex or aggregation pheromones [47,48]. For example, in the Coreidae *Leptoglossus occidentalis* (Heidemann) [48], and *Leptoglosus australis* (Fabricius) [49], the Pentatomidae *Podisus maculiventris* (Say) [50,51], and the Lygaeidae *Oncopeltus fasciatus* Dallas and *Lygaeus kalmii* Stål [52,53] aggregation pheromones are released only by the males. In the Coreidae *Amblypelta lutescens lutescens* Distant [54], the aggregation pheromone is released by both sexes. While in Pentatomidae, *Eurydema rugosa* Motschulsky [55], and *N. viridula* [56] aggregation pheromones are released by the nymphs.

The aggregation pheromone of *L. zonatus* may have a double function as in the *N. viridula* nymphs where the low concentration of the pheromone attracts more individuals but repels them at high concentrations [56]. This idea is supported by the fact that Leal et al. [30] reported aggregations of this insect and unsuccessfully tried to identify the aggregation pheromone but instead reported an alarm pheromone. *Leptoglossus zonatus* males and females have metathoracic scent glands [29] and may release a different concentration of the pheromone depending on the situation. The identification of the aggregation pheromone is mandatory to validate this idea.

The information reported on the attractiveness of some colors to nymphs and adults and the behavioral evidence of an aggregation pheromone in *L. zonatus* unveils the possibility to design a blue-green colored trap lured with the aggregation sex pheromone. The identification of the aggregation pheromone is underway, and we aim to evaluate different colored trap designs for *L. zonatus* in field conditions.
